# UAV-Aided Dual-User Wireless Power Transfer: 3D Trajectory Design and Energy Optimization

**DOI:** 10.3390/s23062994

**Published:** 2023-03-10

**Authors:** Xiaogang Gou, Zhaojie Sun, Kaiyuan Huang

**Affiliations:** 1 The 54th Research Institute of China Electronics Technology Group Corporation, Shijiazhuang 050081, China; 2 The Key Laboratory of Science and Technology on Communications, University of Electronic Science and Technology of China, Chengdu 611731, China

**Keywords:** wireless power transfer, UAV trajectory design, energy optimization

## Abstract

Unmanned aerial vehicles (UAVs) have been widely considered to enhance the communication coverage, as well as the wireless power transfer (WPT) of energy-constrained communication networks to prolong their lifetime. However, the trajectory design of a UAV in such a system remains a key problem, especially considering the three-dimensional (3D) feature of the UAV. To address this issue, a UAV-assisted dual-user WPT system was investigated in this paper, where a UAV-mounted energy transmitter (ET) flies in the air to broadcast wireless energy to charge the energy receivers (ERs) on the ground. By optimizing the UAV’s 3D trajectory toward a balanced tradeoff between energy consumption and WPT performance, the energy harvested by all ERs during a given mission period was maximized. The above goal was achieved through the following detailed designs. On the one hand, on the basis of previous research results, there is a one-to-one correspondence between the UAV’s abscissa and height, so only the relationship between the height and time was focused on in this work to obtain the UAV’s optimal 3D trajectory. On the other hand, the idea of calculus was employed to calculate the total harvested energy, leading to the proposed high-efficiency trajectory design. Finally, the simulation results demonstrated that this contribution is capable of enhancing the energy supply by carefully designing the 3D trajectory of the UAV, compared to its conventional counterpart. In general, the above-mentioned contribution could be a promising way for UAV-aided WPT in the future Internet of Things (IoT) and wireless sensor networks (WSNs).

## 1. Introduction

In recent years, the use of unmanned aerial vehicles (UAVs) has been widely considered for wireless communications, due to their deployment flexibility and controllable mobility [1,2,3]. Specifically, UAVs have been explored to improve the security of wireless information delivery [4], to enhance the wireless connection as a reliable relay [5,6], to break through the energy supply bottleneck of wireless networks [7,8], and to provide a stable and reliable communication framework [9,10]. Meanwhile, UAVs can be well integrated with current pioneering wireless transmission techniques such as reconfigurable intelligent surface (RIS) [11], millimeter-wave (mmWave) [12], and terahertz [13]. Therefore, they are also considered as a flexible new type of base station or relay toward the realization of the sixth-generation networks [14,15]. Furthermore, the Internet of Things (IoT) remains an important scenario for 6G wireless networks [16,17], and the energy supply for long-term work still remains a big problem. Therefore, with the aid of a UAV, while exploring the new design freedom from the air, the new construction of wireless power transfer (WPT) has attracted much attention in order to generate a more efficient and stable energy supply to break through the bottleneck of the IoT network [18,19]. Another advantage lies in that, due to the multi-dimensional moving freedom of the UAV, the original WPT connection can be enhanced by avoiding blockage and shadow fading, so that the transmission channel can be simplified to a line-of-sight (LoS) one thanks to the introduction of the UAV [20,21]. As a result, UAVs can significantly enhance the WPT performance while offering stable supply to the fixed-location energy transmitters (ETs) compared to the conventional WPT systems.

Due to the above-mentioned advantages and potentials, UAV-aided WPT systems have been widely investigated in the literature. For example, the authors in [22] first considered a novel WPT architecture, where UAVs were utilized as mobile ETs to provide wireless energy to charge a number of ERs deployed on the ground. By making full use of the controllable mobility of the UAV, the WPT efficiency of the new WPT system is anticipated to be greatly improved by a rational trajectory design. In addition, positioning and trajectory designs for UAVs to maximize the energy transfer efficiency were also studied in [23,24,25,26,27,28]. Xu et al. [18] maximized the transferred energy amounts towards all ERs during the charging period, and min-energy maximization was considered in [23,24,25] to improve the fairness. Jiang et al. [26], Xie et al. [27], and Cho et al. [28] investigated the cooperative UAV trajectory design and wireless resource allocation problem for simultaneous wireless information and power transfer (SWIPT) systems. However, most of the aforementioned works considered the UAVs flying at a constant altitude in the sky. To efficiently employ the UAV as a WPT source to power ground ERs, a precise three-dimensional (3D) trajectory design must be conducted to meet the needs of the energy-limited ground users.

According to the above-mentioned issue, the most-important design problem in UAV-aided WPT systems is carefully controlling the flying routes with the high demand of energy efficiency. In pursuit of an economic and efficient trajectory design, recently, in [18], the maximization of the sum-energy was achieved through a more efficient trajectory design for the UAV and ground ERs, which indicates that there is a one-to-one mapping of the UAV’s abscissa to the height. Motivated by [18], the UAV’s 3D trajectory was optimized to maximize the amount of transferred energy at all receivers during the charging period in this paper.

Different from previous works, it was assumed that the UAV flies within the height range of $\left[H_{0},H_{\max}\right]$, where $H_{0}$ is the height of the charging pile and $H_{\max}$ is the target height of the UAV. The main research contributions are described as follows:

- This paper gives a brief description of the so-called problem of maximizing the sum-energy for enhancing the energy supply. That is to say, this work focused on maximizing the achievable energy for all ERs during a given charging period by optimizing the UAV’s 3D trajectory.
- This work further optimized the relationship between the UAV’s altitude and time to obtain the UAV’s 3D trajectory according to the results in [18]; in addition, the total harvested energy was calculated with the idea of calculus.
- The theoretical and numerical results demonstrated that the proposed design outperformed the benchmark schemes in terms of higher energy transferred to all ERs.

On the one hand, the UAV’s 3D trajectory was efficiently optimized compared to the current literature, so as to make a balanced tradeoff between energy harvesting and consumption. On the other hand, after a careful design, the energy harvested by all ERs during a given mission period was maximized for WPT.

The remainder of this paper is organized as follows. The detailed system model and general sum-energy maximization problem for the UAV-aided WPT are introduced in Section 2. In Section 3, the optimization problem of the 3D trajectory design of the UAV-aided WPT system is explored. Finally, numerical results are presented in Section 4 to demonstrate the effectiveness of the proposed method, and the paper is concluded in Section 5.

## 2. System Model

**Figure 1:**
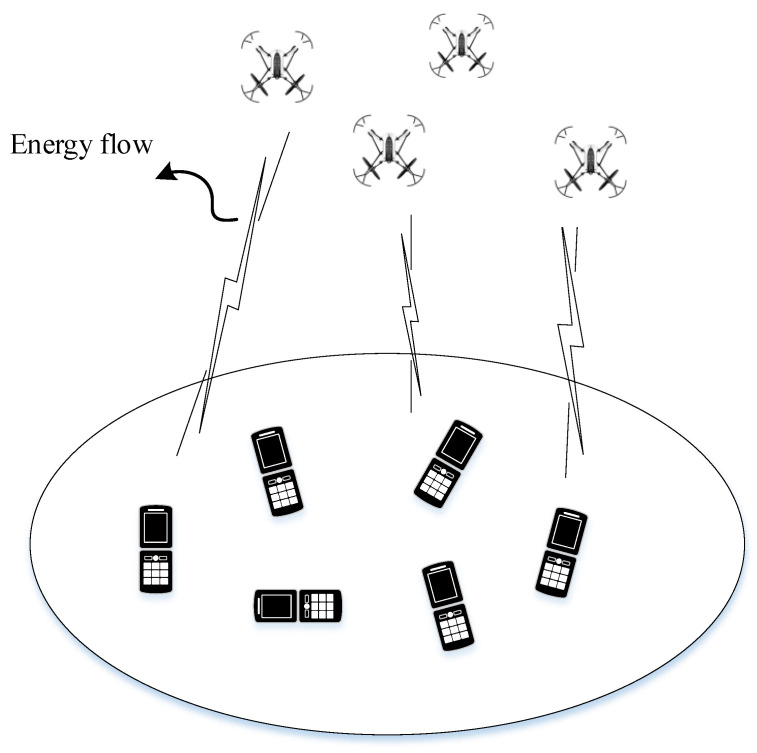
The system model of UAV-aided WPT.

In general, the UAV-aided WPT system can be described by Figure 1. For some special transmission environments such as the IoT, the energy of the device is limited due to the size and the stationary of the device. Then, in the considered system, the UAV serves as a special energy supply for the users. Considering the freedom of space, the 3D trajectory was developed. In the context of a UAV-aided dual-user wireless power transmission system, the UAV was utilized to deliver the energy to two energy-limited nodes. Each ER, k=1,2, has an unchanged location $\left(x_{k},y_{k},0\right)$ on the ground, which is a priori known to the UAV to facilitate its trajectory design. We focused on a given mission period with duration *T*, which generally depends on the energy stored by the UAV. The UAV was assumed to fly within the altitude range $\left[H_{0},H_{\max}\right]$, so that the path for the wireless power transmission between the UAV and ER was, respectively, assumed to be an LoS channel. As a result, this paper can adopt the traditional LoS channel model in the free space as in [22,23,24]. At time instant *t*, the UAV’s time-varying location is denoted as $\;\left(x\left(t\right),y\left(t\right),h\left(t\right)\right)$. As a result, the channel power gain from the UAV to ER *k* can be modeled as

(1) $$h_{k}\left(t\right)=\beta_{0}d_{k}^{-2}\left(t\right),$$

where

(2) $$d_{k}\left(t\right)=\sqrt{{\left(x\left(t\right)-x_{k}\right)}^{2}+{\left(y\left(t\right)-y_{k}\right)}^{2}+h^{2}\left(t\right)}$$

stands for the distance from the UAV to each independent ER, while $\beta_{0}$ is represented by the power of the transmission channel gain built on a reference distance as $d_{0}=1$ m. When the transmit power of the UAV is fixed to be *P*, at each time *t*, the power harvested by ER *k* can be written as $Q_{k}\left(x\left(t\right),y\left(t\right),h\left(t\right)\right)$ and can be further expressed as

(3) $$\begin{array}{c} Q_{k}=\eta h_{k}\left(t\right)P\\ =\frac{\eta \beta_{0}P}{{\left(x\left(t\right)-x_{k}\right)}^{2}+{\left(y\left(t\right)-y_{k}\right)}^{2}+h^{2}\left(t\right)} \end{array}$$

where η is a real number between 0 and 1, standing for the efficiency of the energy delivery and conversion at each side of the ER. Therefore, based on the UAV’s trajectory $\left\{x\left(t\right),y\left(t\right),h\left(t\right)\right\}$ which was the optimization objective of this paper, the sum-energy harvested through ER *k* during the whole UAV-aided WPT period *T* is written as $E_{k}\left(\left\{x\left(t\right),y\left(t\right),h\left(t\right)\right\}\right)$ and is further expressed as

(4) $$E_{k}=\int_{0}^{T}Q_{k}dt.$$

Then, based on the above model, the design goal of this paper was to maximize the energy harvested from all the ERs through optimizing the trajectory of the UAV as $\left\{x\left(t\right),y\left(t\right),h\left(t\right)\right\}$. Specifically, the so-called sum-energy maximization process can be summarized as

(5) $$\max_{\left\{x\left(t\right),y\left(t\right),h\left(t\right)\right\}}\underset{k}{\Sigma }\int_{0}^{T}Q_{k}dt$$

Although the above model could be a representation of the UAV-aided WPT design, the optimal theoretical solution may still be hard to achieve, since the harvested energy $E_{k}$ in (4) is a non-concave function of the UAV’s trajectory. Therefore, the solution to the problem in (5) is also a non-convex optimization one that cannot be easily solved using traditional ways. Therefore, in order to address this issue, this work utilized the results in [18], so there is a parallel relation between the UAV’s abscissa and height. Consequently, only the relationship between the height and time need to be optimized to obtain the optimal UAV 3D trajectory.

## 3. Optimal Trajectory Design

It is noted that setting the ordinate of the UAV as $y\left(t\right)\;=0,\;t\in \left[0,T\right]$ is reasonable, which would greatly simplify the optimization problem. Additionally, according to the analysis in [18], $x\left(t\right)$ can be viewed as a function of the height $h\left(t\right)$, which can be expressed as

**Figure 2:**
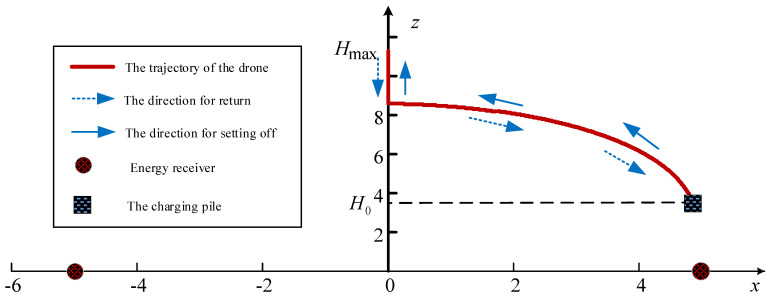
The trajectory design for a two-user WPT system.

**Figure 3:**
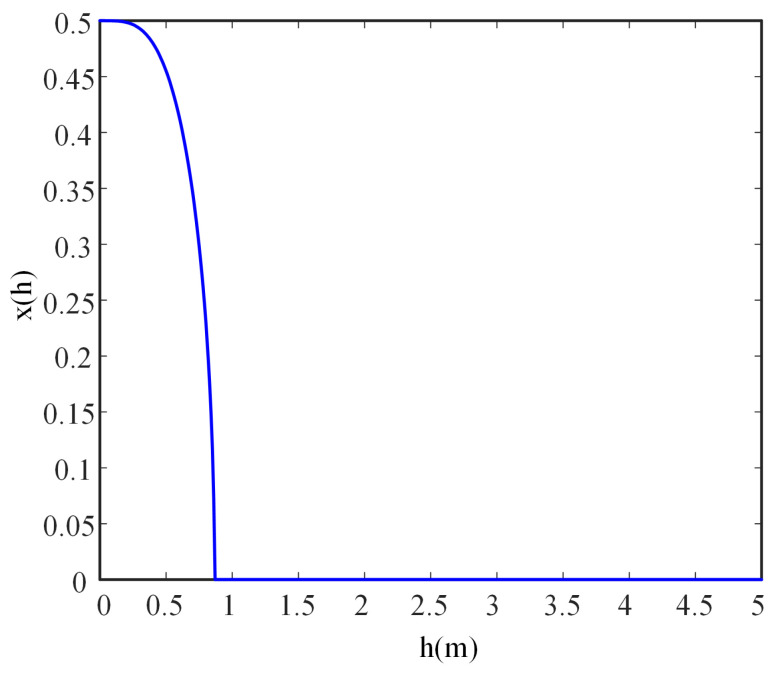
The relationship between the UAV’s abscissa and height.

(6) xh=D44+h2D2−D24+h2,if0≤h≤3D3D220,if3D3D22≤h≤Hmax

where *D* is the distance between two ERs. According to (6), the UAV’s flight trajectory diagram can be drawn as shown in Figure 2. At the same time, the relationship between the UAV’s abscissa and height is shown in Figure 3. Assuming Hmax>3D3D22, the mission period *T* is long enough and the UAV flies at a constant velocity *V*. Suppose that the UAV reaches the height of $H_{\max}$ at time $\hat{t}$ and hovers there for a period of $T-2\hat{t}$, then it would return to the initial point at the speed of *V* for charging and waiting for the next mission.

Obviously, the trajectory shown in Figure 2 consists of a straight line and a curve. Firstly, the idea of calculus can be adopted to obtain the length of the curve. Specifically, dividing the interval h∈H0,3D3D22 into *N* equal parts, the step size φ is expressed as

(7) φ=3D3D22−H0N.

**Figure 4:**
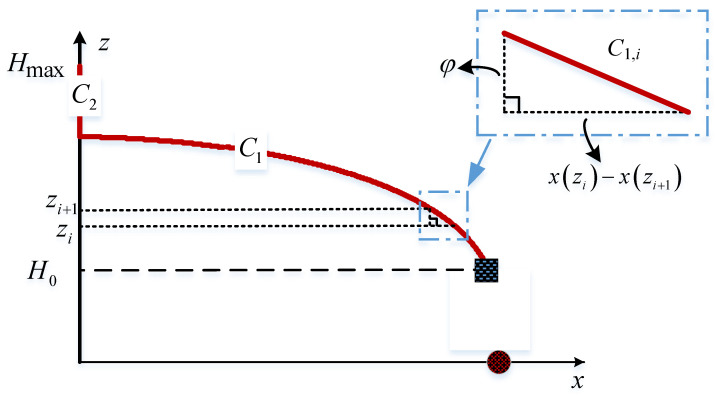
Calculating the received energy with calculus.

This paper denotes the *i*-th interval as $\left[z_{i-1},z_{i}\right]$, where $z_{i}=H_{0}+\varphi i,i=1,\cdots ,N$ and $z_{0}=H_{0}$. As shown in Figure 4, when the step size φ is small enough, the flight path corresponding to $\left[z_{i-1},z_{i}\right]$ can be approximated as a straight line, whose length can be calculated by the triangle marked in Figure 4 with the formula $C_{1,i}=\sqrt{{\left(x\left(z_{i-1}\right)-x\left(z_{i}\right)\right)}^{2}+\varphi^{2}}$. Additionally, the flight period over it is t1,i=C1,iC1,iVV, during which, assuming that the UAV hovers at position $\left(x\left(z_{i}\right),0,z_{i}\right)$, then the total energy collected during this period is

(8) Q1,i=ηβ0Pt1,i1xzi−DD222+zi2+1xzi+DD222+zi2.

As a consequence, the total length of the curve is

(9) $$\begin{array}{cc} C_{1} & =\lim_{N\to \infty }\sum_{i=1}^{N}C_{1,i}\\ & =\lim_{N\to \infty }\sum_{i=1}^{N}\sqrt{{\left(x\left(z_{i-1}\right)-x\left(z_{i}\right)\right)}^{2}+\varphi^{2}}, \end{array}$$

the total collected energy is

(10) Q1=limN→∞∑i=1NQ1,i=ηβ0PlimN→∞∑i=1Nt1,i1xzi−DD222+zi2+1xzi+DD222+zi2

and the time for the UAV to reach point 0,0,3D3D22 is

(11) t1=limN→∞∑i=1Nxzi−1−xzi2+φ2xzi−1−xzi2+φ2VV.

Note that the length of the straight line is

(12) C2=Hmax−3D3D22

and the time required is

(13) t2=Hmax−3D3D22Hmax−3D3D22VV.

Suppose that the UAV rises a distance from 0,0,3D3D22 with time τ, then the coordinate of the UAV is 0,0,3D3D22+Vτ, and the total received power of the two energy receivers is expressed as

(14) Preceiveτ=2ηβ0PD2D244+3D3D22+Vτ2

Therefore, the total energy received by the two energy receivers when the UAV rises from 0,0,3D3D22 to $\left(0,0,H_{\max}\right)$ is expressed as

(15) Q2=∫0t2Preceiveτdt=2ηβ0P∫0t21D2D244+3D3D22+Vτ2dτ=t=3D3D22+Vτ2ηβ0P∫3D3D223D3D22+Vt21D2D244+t2dt−3D3D22V=2ηβ0PV1DD22arctantDD223D3D223D3D22+Vt2=4ηβ0PVDarctan2HmaxD−π3

Finally, the UAV would hover at $\left(0,0,H_{\max}\right)$ for a period of $\left(T-2\left(t_{1}+t_{2}\right)\right)$. During this period, the total energy collected by the energy receivers is written as

(16) Q3=2ηβ0PT−2t1+t2D2D244+Hmax2

To sum up, in the case of sufficient mission period *T*, the total energy obtained by the energy receiver is

(17) $$Q_{3D}=Q_{1}+Q_{2}+Q_{3}$$

During the mission period *T*, for any time $t\in \left[0,T\right]$, the UAV’s coordinate under the optimal trajectory is given by

(18) loc=xH0+3D3D22−H0Ni,0,H0+3D3D22−H0Ni,t∈0,t10,0,3D3D22+Vt−t1,t∈t1,t1+t20,0,Hmax,t∈t1+t2,T−t1+t2xHmax−Vt+t1+t2−T,0,Hmax−Vt+t1+t2−T,t∈T−t1+t2,T−t1xH0+3D3D22−H0Ni,0,H0+3D3D22−H0Ni,t∈T−t1,T

As theoretically derived in the above, for UAV-aided WPT systems, the UAV can obtain an optimal trajectory as a balanced tradeoff between the energy efficiency and the overall performance. In the next section, the simulation results will be given to prove the effectiveness of the designed 3D trajectory. The details are summarized in Algorithm 1.
**Algorithm 1:** UAV trajectory design algorithm.**Require:** Initialize η, $\beta_{0}$, *P*,$(x\left(z_{i}\right),0,z_{i}),i=1,\cdots ,N$, $H_{0}$, $H_{max}$, *v*      **Output:**
$Q_{1}$, $Q_{2}$, $Q_{3}$, loc
1:Compute φ, $t_{1}$, $t_{2}$ with (7), (11), and (13), respectively.2:Update the collected energy $Q_{1}$, $Q_{2}$, and $Q_{3}$ with (10), (15), and (16), respectively.3:Find the optimal trajectory design loc for the optimization problem in (5).


## 4. Numerical Results

In this section, the wireless energy transmission performance of the two-user UAV-enabled WPT system is provided to verify the effectiveness of the 3D UAV path-planning algorithm proposed in Section 3. The specific simulation parameters are shown in Table 1 and Table 2 to describe the transmission environment.

**Figure 5:**
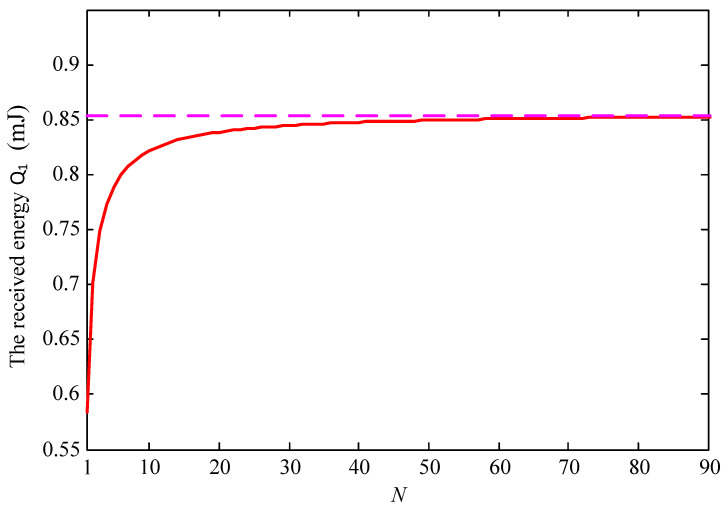
Convergence analysis of the proposed scheme.

For the above-described configuration, Figure 5 exhibits the convergence analysis of the total collected energy $Q_{1}$ shown in Formula (10). In the simulation, let $H_{0}=0.5$ m, $H_{\max}=5$ m, D=1 m, and V=0.5 m/s. The relationship between the average received energy and *N* is drawn to validate that employing calculus to obtain the harvested energy is feasible, because when *N* is large enough, the average received energy tends to be a constant. In this case, enough energy supply is expected to support the devices, which is important for energy-limited networks such as the IoT. However, this additional energy supply is achieved at the cost of the energy of the UAV.

**Figure 6:**
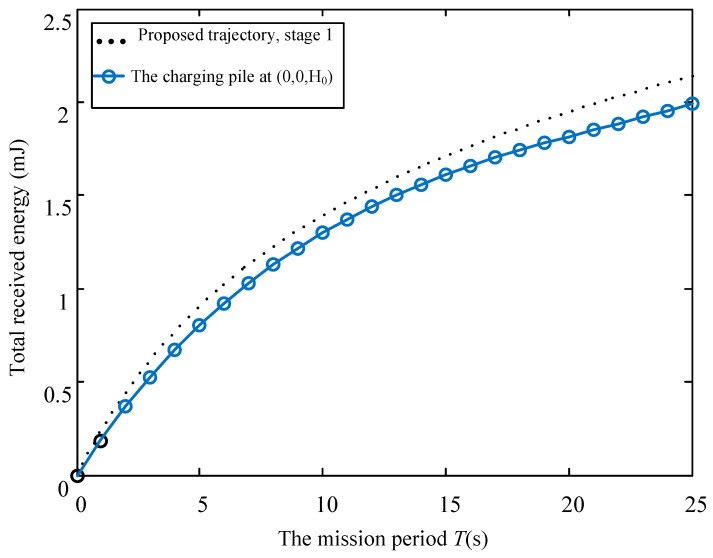
The relationship between total received energy and mission period *T* with *V* = 0.2.

**Figure 7:**
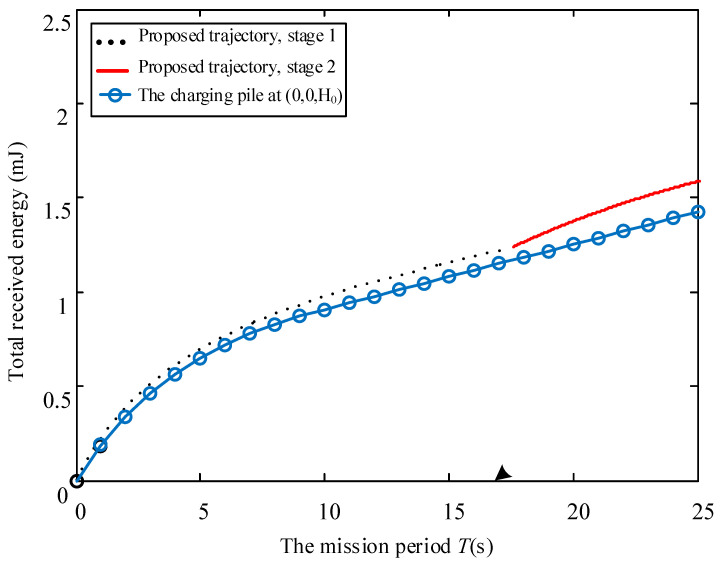
The relationship between total received energy and mission period *T* with *V* = 0.4.

**Figure 8:**
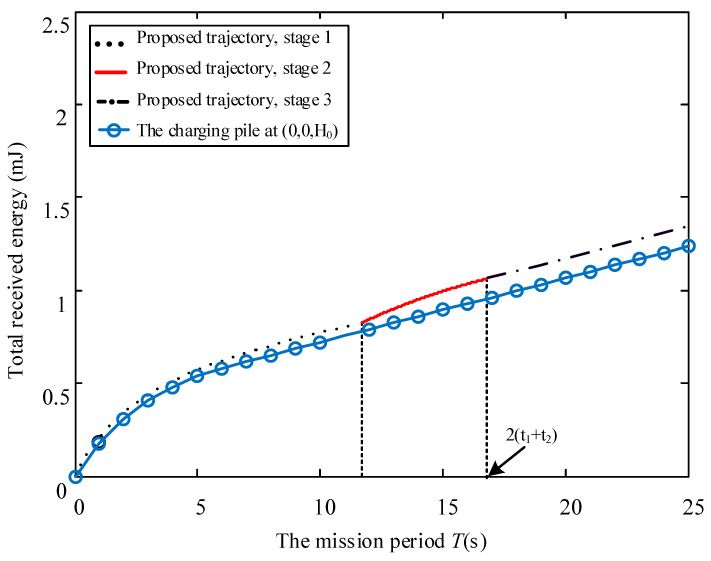
The relationship between total received energy and mission period *T* with *V* = 0.6.

**Figure 9:**
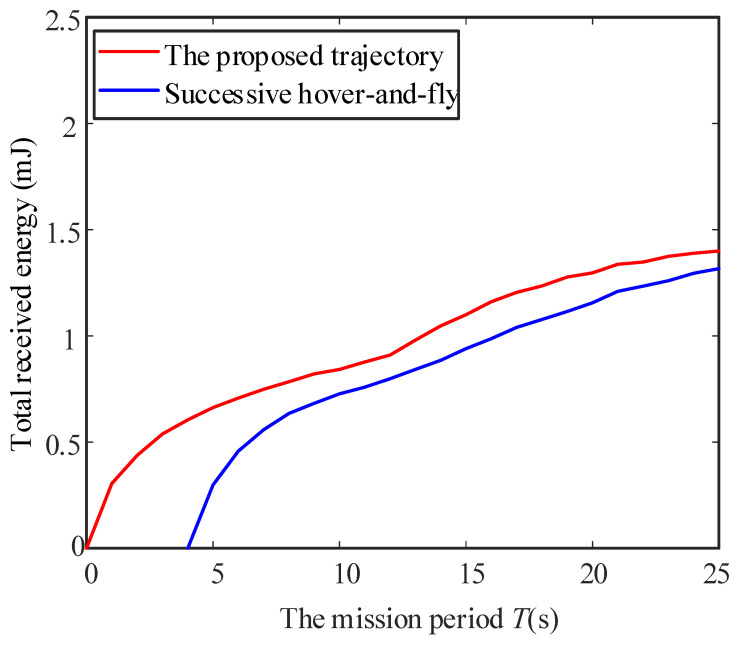
The total received energy between the proposed optimal path algorithm and the successive hover-and-fly trajectory scheme.

Considering different mobile speed V, Figure 6, Figure 7 and Figure 8 disclose the relationship between the total energy received by the ERs and the mission period *T*. Suppose that the process of the UAV flying over curves $C_{1}$ and $C_{2}$ and hovering at the highest place is denoted as Stage 1, Stage 2, and Stage 3, respectively. It is observed from the figures that the total received energy increases with the task period *T*, as expected. Specifically, when V=0.2 m/s, we arrive at $2t_{1}\geq T$ in Figure 6, which denotes that the UAV starts to return to the starting point before it reaches directly above the midpoint of the two energy receivers. Therefore, the UAV will not go through Stage 2 and Stage 3. Furthermore, when V=0.4 m/s in Figure 7, we arrive at $2t_{1}<T\leq 2\left(t_{1}+t_{2}\right)$, which denotes that the UAV continues to rise after reaching the position 0,0,3D3D22, but returns before reaching the highest altitude. When V=0.6 m/s in Figure 8, we arrive at $2\left(t_{1}+t_{2}\right)<T$, and the UAV reaches the highest point and hovers here for a period of $T-2\left(t_{1}+t_{2}\right)$, then starts to return. As can be seen from the figures, the curve corresponding to the third stage is a straight line with a slope of 2ηβ0P2ηβ0PD2D244+Hmax2D2D244+Hmax2, which is consistent with the conclusion in (16). In addition, this work also provides the total energy received by the system when the charging pile is built at the point $\left(0,0,H_{0}\right)$ to compare the performance. In a nutshell, we demonstrated that the method proposed in this paper achieved significant performance gains. In Figure 9, the total received energy is compared between the proposed optimal path algorithm and the successive hover-and-fly trajectory scheme [18], which is only set as the locations above all ERs, rather than optimizing the UAV’s hovering locations during a given mission period. It was observed that the proposed trajectory design provides better performance than the successive hover-and-fly path.

Through the theoretical derivation and simulation results of this contribution, this paper demonstrated that the UAV can be extended to WPT systems to support energy-limited users with IoT sensors and low-cost devices. That is to say, the UAV may be a candidate energy supplier for the IoT to maintain its work. However, this advantage comes at that cost of additional energy consumption at the UAV side, which should be carefully considered for a balanced tradeoff through the trajectory design for the UAV. Considering the space freedom offered by the air, a more flexible 3D trajectory design is effective for such a system.

## 5. Conclusions

In this work, the UAV 3D trajectory optimization problem was investigated to maximize the total received energy in a wireless system, based on the assumption that the UAV is dispatched to charge two ground nodes through radio frequency wireless power transfer. The main contribution of this work can be summarized as follows. On the one hand, the optimization problem was ultimately solved by optimizing the relationship between the height and time according to the one-to-one correspondence between the UAV’s abscissa and height, through theoretical derivation. On the other hand, the sum-received-energy among all ERs was modeled and then calculated by calculus to arrive at an accurate solution. Finally, the numerical results demonstrated that the maximal attainable energy was increased with the proposed design. Specifically, compared to conventional counterpart [18], an energy increase of 20% can be achieved through the proposed 3D trajectory design.

## Figures and Tables

**Table 1:** Summary of main notations.

Symbol	Definition
*t*	Time
$\;\left(x\left(t\right),y\left(t\right),h\left(t\right)\right)$	The UAV’s time-varying location at time instant *t*
$h_{k}\left(t\right)$	The channel power gain from the UAV to ER *k*
$Q_{k}$	The power harvested by ER *k*
$E_{k}$	The sum-energy harvested through ER *k* during the whole UAV-aided WPT period *T*
ϕ	Step size

**Table 2:** Simulation parameters of two-user WPT system.

Parameter	Value
UAV’s transmit power	40 dBm
UAV’s height	5 m
Channel gain	−30 dB
Safe distance	1 m

## Data Availability

Not applicable.
